# Progress in understanding the diagnostic and pathogenic role of autoantibodies associated with systemic sclerosis

**DOI:** 10.1097/BOR.0000000000000325

**Published:** 2016-09-28

**Authors:** May Y. Choi, Marvin J. Fritzler

**Affiliations:** Cumming School of Medicine, University of Calgary, Calgary, AB, Canada

**Keywords:** autoantibodies, clinical care pathway, early systemic sclerosis, functional autoantibodies, pathogenesis

## Abstract

**Purpose of review:**

At the time of diagnosis, systemic sclerosis (SSc) is often well established with significant irreversible tissue and organ damage. Definitions of ‘early SSc’ have been proposed, which include the presence of SSc-associated autoantibodies. In addition, functional autoantibodies that are believed to be involved in SSc pathogenesis need to be considered. In this review, recent advances in the diagnostic utility and pathogenic role of autoantibodies in early SSc are summarized. Moreover, we propose a clinical care pathway illustrating how autoantibody testing along with key clinical features can be used to make an earlier diagnosis of SSc.

**Recent findings:**

Recent evidence has helped to develop a clearer understanding of the natural history, early clinical features, and autoantibodies that are predictors of SSc. The role of functional autoantibodies is leading to innovative approaches to evidence-based interventions and therapies that are based on mechanisms of disease.

**Summary:**

Despite substantial advances, the high morbidity and mortality that currently characterizes SSc can largely be attributed to a delay in diagnosis, gaps in our understanding of the role of autoantibodies in early disease, and limited effective therapeutic options. An early and accurate diagnosis of SSc and use of autoantibody testing embedded in evidence-based clinical care pathways will help improve SSc-associated clinical outcomes and healthcare expenditures.

## INTRODUCTION

Systemic sclerosis (SSc) is a chronic, multisystem disorder that evolves through stages of early initiation (triggering events), disease amplification, and later progression, all characterized by an overlapping triad of autoimmunity, microvascular abnormalities, and variable degrees of fibrosis [1^▪▪^]. Greater than 85% of established SSc patients have circulating autoantibodies directed to intracellular and extracellular targets [2,3^▪^]. Historically, autoantibodies directed to nuclear components [antinuclear antibodies (ANAs)] were the first to be described only to be followed by an appreciation that cytoplasmic, cell membrane, and even extracellular components were included in the SSc B-cell repertoire [2,3^▪^,^4^]. In addition to their role as diagnostic biomarkers, there is increasing evidence that autoimmunity occurs early in disease, plays an important role in pathogenesis, and is correlated with end-organ manifestations [4,5^▪^].

To date, there is limited evidence as to the primary causes of SSc or the molecular mechanisms underlying its clinical onset, progression, and outcomes [1^▪▪^]. An etiopathogenic model integrates four features of the disease: inherent susceptibility (e.g. genetic and environmental factors); early initiation with triggering events (e.g. chemical, neoplastic, infections, endocrine); amplification (e.g. severity genes and immunologic factors); and later progression (e.g. secondary pathology and internal organ complications). Importantly, the progression of disease is likely not sequential as commonly thought, but rather there is simultaneous dysfunction in normal regulatory mechanisms of endothelial physiology, immune tolerance, and extracellular matrix turnover. There is also an emerging evidence supporting a pathogenic role for certain autoantibodies (e.g. ‘functional autoantibodies’) [6^▪^,^7^^▪▪^]. Therefore, advances in understanding autoinflammatory pathways and T/B-cell activation in early SSc [1^▪▪^,^8^] can present important therapeutic implications [9,10].

SSc is one of the most disabling ANA-associated rheumatic diseases [AARDs: SSc, systemic lupus erythematosus, autoimmune inflammatory myopathies, mixed connective tissue disease (MCTD), Sjögren's syndrome], severely affecting the quality of life [11] and attended by significant healthcare expenditures [12–15]. In addition, early SSc patients may be categorized as undifferentiated connective tissue disease (UCTD) [16^▪▪^] or MCTD [17], and by the time a diagnosis of definite SSc is made, the effectiveness of conventional therapies is limited because the patient already has excess collagen and other extracellular matrix deposition and remodeling of the skin and internal organs and associated serious complications [18]. Hence, a clearer understanding of SSc pathogenesis in early phases of the disease is critical to achieve an early and accurate diagnosis and then evidence-based effective treatment. This review will focus on the recent advances in understanding the importance of early diagnosis and on SSc autoantibodies and their clinical and pathogenic relevance. We propose a clinical care pathway highlighting the use SSc autoantibodies and key clinical features to help with the diagnosis and management of early disease. 

**Box 1:**
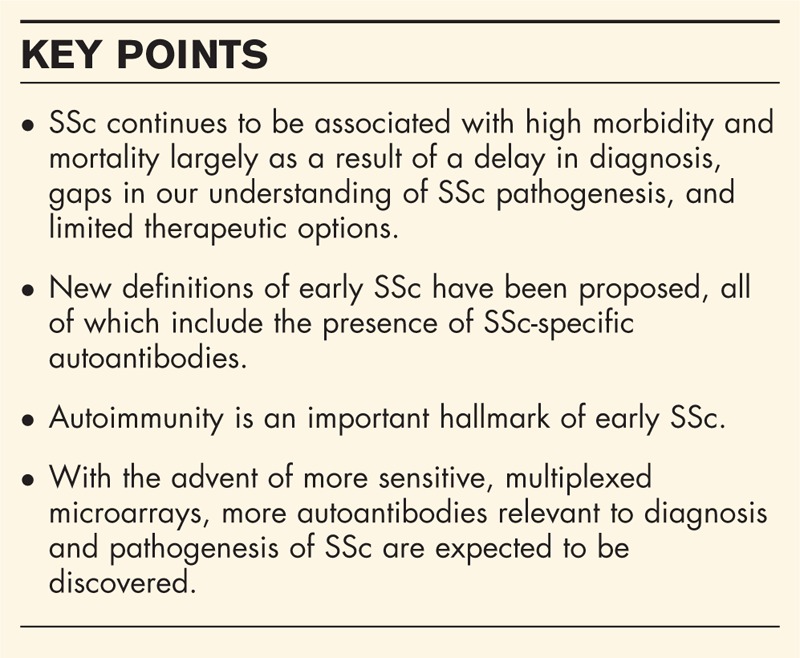
no caption available

## EARLY SYSTEMIC SCLEROSIS

There is mounting anticipation that an earlier diagnosis of SSc may allow interventions that could block or slow the progression of disease [19^▪^,20^▪^]. One of the limitations of the 1980 American College of Rheumatology (ACR) criteria [21] is that it depended on features that are the sequelae of the disease, therefore, limiting the ability to detect early disease [22]. The ACR-European League Against Rheumatism (EULAR) developed a revised classification criteria with better sensitivity and specificity (0.91 and 0.92, respectively) compared with the 1980 ACR criteria (0.75 and 0.72, respectively) [23]. However, some patients such as those with Raynaud's phenomenon, an SSc autoantibody, and abnormal capillaroscopy would still not be classified as SSc. To help identify patients with early SSc, several definitions and an approach to an early diagnosis of SSc have been proposed (Table 1) [24–26,27^▪▪^].

**Table 1:** Definitions of early systemic sclerosis

Definition	Year	Criteria	Antibodies
Leroy and Medsger criteria [24]	2001	Limited SSc or early SSc	ACENP
		RP (objective documentation) plus any one of these:	ATA
		SSc-type nailfold capillary pattern	Anti-U3RNP
		SSc-selective autoantibodies	Anti-PM-Scl
		OR	Anti-fibrillin
		RP (subjective only) plus both:	Anti-RNAP I or III
		SSc-type nailfold capillary pattern	
		SSc-selective autoantibodies	(titer ≥1 : 100)
		Limited cutaneous SSc:	
		Criteria for limited SSc plus cutaneous changes distal to elbows, knees, and clavicles	
		Diffuse cutaneous SSc:	
		Criteria for limited SSc plus proximal cutaneous	
Nadashkevich *et al.* [25] ABCDCREST	2004	Three or more criteria of:	ACENP
			ATA
		**A**ntibodies	Anti-fibrillarin
		**B**ibasilar pulmonary fibrosis	
		**C**ontractures of the digital joints or prayer sign	
		**D**ermal thickening proximal to the wrists	
		**C**alcinosis cutis	
		**R**P (at least two phase color change)	
		**E**sophageal distal hypomotility or reflux esophagitis	
		**S**clerodactyly or non-pitting edema of the fingers	
		**T**elangiectasia	
Very early diagnosis of systemic sclerosis or VEDOSS [26]	2011	Criteria considered as having a high clinical relevance for the VEDOSS:	ACENP
		RP	ATA
		Puffy fingers turning into sclerodactyly	
		Abnormal capillaroscopy with scleroderma pattern	
		Antibodies	
		Criteria considered as leading to an early referral:	
		RP	
		Puffy fingers	
		Positive ANA	
Undifferentiated connective tissue disease [16^▪▪^]	1980	Unclassifiable systemic autoimmune diseases that share clinical and serological manifestations with definite AARD	Any AARD-related autoantibody

AARD, antinuclear antibodies-associated rheumatic disease; ACENP, anticentromere antibody; ANA, anti-nuclear antibody; ATA, antitopoisomerase I; PM/Scl, polymyositis/scleroderma antigen; RNAP, RNA polymerase; RNP, ribonucleoprotein; RP, Raynaud's phenomenon; SSc, systemic sclerosis.

LeRoy and Medsger [24] first defined ‘early SSc’ as patients with Raynaud's phenomenon and SSc autoantibodies and/or typical SSc nailfold capillaroscopic findings. This criterion was validated by a long-term follow-up of ‘early SSc’ patients over 20 years [28,29^▪^]; however, a more recent study [30] revealed that only 35% of ‘early SSc’ patients satisfied the 2013 ACR/EULAR classification criteria. As not all patients progress to overt SSc, this definition may not accurately capture truly early SSc patients. In 2004, Nadashkevich *et al.*[25] proposed another classification criteria called ‘ABCDCREST’ [**A**utoantibodies to CENP, Scl-70/topo I, or fibrillarin; **B**ibasilar pulmonary fibrosis; **C**ontractures of the digital joints or prayer sign; **D**ermal thickening proximal to the wrists; **C**alcinosis cutis; **R**aynaud's phenomenon (RP); **E**sophageal distal hypomotility or reflux-esophagitis; **S**clerodactyly or non-pitting digital edema; **T**elangiectasia] aimed to increase sensitivity of the ACR 1980 classification criteria [21] in part by including patients with early disease.

A ‘very early diagnosis of systemic sclerosis’ (VEDOSS) includes criteria that were proposed and validated by the EULAR Scleroderma Trial and Research group [26,31]. The VEDOSS criteria take into consideration features that have high clinical relevance and would prompt an early referral. Recent studies reveal that VEDOSS patients, especially if they already have digital ulcers [32], can already have significant internal organ involvement including interstitial lung disease [33] and esophageal and anorectal disorders [34]. Hence, there is a need to diagnose SSc as early as possible and assess for organ involvement even in the early stages of disease.

Some clinicians may classify early SSc as UCTD. UCTD is a term that refers to patients who have unclassifiable systemic autoimmune diseases that share clinical and serological manifestations with definite AARD [16^▪▪^]. UCTD patients may either remain as ‘stable UCTD’ or represent an ‘early phase’ of CTD. In a 5-year follow-up of UCTD patients, 35% progressed to a specific CTD but only 2.1% progressed to SSc. Although 65% remained as UCTD and 12% achieved complete remission, almost 80% had developed major organ involvement [35]. The highest probability of progression to a defined CTD was within 2 years after onset, and the presence of autoantibodies was the most important predictor of faster progression to SSc in UCTD patients, particularly in those with preclinical internal organ involvement at baseline [30]. The limitations of such studies are that the natural history of UCTD is largely unknown and it could be argued that the patients who do not evolve to an AARD are those that have received effective or protective therapies.

Akin to UCTD, it has been reported that the majority of MCTD patients eventually evolve into another AARD such as SSc [17,36]. However, more recent studies, including a longitudinal study [17] of 50 incident MCTD patients from Olmstead County, USA observed that only 4% evolved into SSc. In this study, it was suggested that when studies of MCTD adhere to classification criteria, the progression of MCTD to AARD is uncommon. However, as with UCTD, there are several confounding issues that need to be considered: there are at least four different criteria used for classification of MCTD [17]; treatment of MCTD may differ from center to center and may have changed from earlier studies; and the possible protective role of anti-U1 ribonucleoprotein (RNP) autoantibodies, a serological hallmark and criteria for the disease [17]. The prevalence of anti-U1RNP in SSc is 2–14% [2,37]. It was reported that autoantibodies that coexist with anti-U1RNP in MCTD sera were predictors of evolution to other AARD [38]. In a recent prospective study [39] of CTD-associated pulmonary arterial hypertension (PAH), which included SSc-associated PAH, anti-U1RNP positive patients were younger and less functionally impaired. Hence, it was suggested that anti-U1RNP might have a protective effect in SSc-associated PAH, although these findings were not statistically significant [hazard ratio 0.47 (95% confidence interval: 0.20–1.11), *P* = 0.085]. Nevertheless, it is interesting that the protective role of anti-U1RNP autoantibodies is a recurring theme. If pre-SSc (UCTD, MCTD) do not progress to full blown disease, it is imperative that research clarifies the protective factors, including autoantibodies [40], that limit the disease expression.

Another facet of SSc is the unique clinical and serological features of SSc-overlap syndromes (SSc-OS) in patients who present with SSc and at least one other CTD at the same time [41]. In a prospective study of 3240 patients registered in the German Network for Systemic Scleroderma and followed for 10 years, 10% were diagnosed as SSc-OS (included patients with MCTD). Of note, the SSc-OS patients often had non-SSc-specific autoantibodies (68.0%; *P* < 0.0001) such as those directed against U1RNP, PM/Scl, SSA/Ro (Sjögren's syndrome A/Ro60 antigen), SSB/La (Sjögren's syndrome antigen B/La antigen), and Jo-1 (histidyl tRNA synthetase). The SSc-OS patients developed musculoskeletal involvement earlier and more frequently and the onset of lung fibrosis and heart involvement was significantly earlier than in patients with limited cutaneous SSc but occurred later than in patients with diffuse cutaneous SSc. The esophagus, kidney, and PAH progression was similar to limited cutaneous SSc patients, whereas diffuse cutaneous SSc patients had a significantly earlier onset. Unfortunately, this study did not distinguish between anti-Ro60/SSA and anti-Ro52/TRIM21 autoantibodies (the second most common autoantibody observed in SSc cohorts) because the latter have recently been associated with interstitial lung disease in SSc [42,43], MCTD [44], and other CTD [45]. Clearly, autoantibody profiles, particularly those that are not SSc-specific, are key distinguishing features of SSc-OS and SSc-OS should likely be regarded as a separate SSc subset.

In the following sections, we describe recent advances in some of the SSc-specific and functional autoantibodies that have roles in the diagnosis of early SSc and pathogenesis, respectively. Preliminary studies have found both classes of autoantibodies to also serve as a basis for clinical phenotypes. We summarize the clinical relevance of these autoantibodies with particular attention to recently published data. For more comprehensive reviews refer to [2,3^▪^,^7^^▪▪^,^46^].

## SYSTEMIC SCLEROSIS AUTOANTIBODIES IN THE DIAGNOSIS OF EARLY DISEASE

Autoantibodies in SSc are believed to be triggered by antecedent vasculopathy or neoplasia, on the background of genetic predisposition and environmental exposures [1^▪▪^]. In the case for antecedent vasculopathy, several SSc-associated autoantigens are fragmented by reactive oxygen species in the setting of ischemia–reperfusion injury, producing immunogenic peptides that are capable of breaking self-tolerance. Therefore, despite their appearance very early in the disease and an initiating feature of SSc, autoantibodies may be sequelae of SSc disease vasculopathy.

SSc autoantibodies, particularly anticentromere protein autoantibodies (ACENP) and antitopoisomerase I (ATA; anti-Scl-70), have been part of every diagnostic criteria of early SSc published thus far. Studies [28,47] have shown that they are strong predictors of progression from isolated Raynaud's phenomenon to SSc. In the ACR-EULAR 2013 classification criteria for SSc as well, the odds ratio of ATA for SSc was 25, ACENP was 14, and anti-RNA polymerase III antibody was 75, relative to other diseases [23]. Several other autoantibodies have also been described (Table 2).

**Table 2:** Frequency and clinical associations of systemic sclerosis autoantibodies in early systemic sclerosis

Antibody	Definition	% Frequency in early SSc^a^	% Frequency in VEDOSS^b^	Clinical association in early SSc^a^ or VEDOSS^b^
Antitopoisomerase I	Antibody to topoisomerase I. Also known as anti-Scl70	12.3–22.5	19.1–22	N/R
Anticentromere antibodies	Antibody to centromere proteins A to F	42.5–67.5	53.6	Predictor of enlarged capillaries and slow rate of microvascular damage [28]
Anti-RNA polymerase I, II, and III	Antibody to RNA polymerases	0–31.3	N/R	Predictor of capillary loss and fast rate of microvascular damage capillaries [28]
Anti-Th/To	Antibody to ribonucleoprotein complexes	15	N/R	Predictor of enlarged capillaries and intermediate rate of microvascular damage [28]

N/R, not reported; SSc, systemic sclerosis.

^a^LeRoy and Medsger's [24] criteria for early SSc.

^b^VEDOSS or very early diagnosis of systemic sclerosis [26].

In a landmark study by Koenig *et al.*[28], 586 consecutive patients with Raynaud's phenomenon and no definite CTD were referred for nailfold capillaroscopy and followed for 20 years. In that time, 12.6% of patients developed definite SSc using the 1980 ACR classification criteria [21] with the majority being patients who were classified as having ‘early SSc’ as per LeRoy and Medsger's criteria [24]. The presence of a SSc autoantibody (ACENP, ATA, anti-Th/To, or anti-RNA polymerase III) and abnormal nailfold capillaries at baseline increased likelihood of developing definite SSc by 60-fold, whereas their absence at baseline practically ruled out this outcome (negative predictive value 98%). The presence of SSc autoantibodies conferred an eight-fold increased risk (adjusted hazard ratio 8.5). In other studies, early SSc autoantibody-positive patients, particularly those with preclinical internal organ involvement at baseline, progressed faster than autoantibody-negative patients [30] and are also at higher risk of fibrotic organ complications [48]. Apart from the most common SSc autoantibodies (ACENP, ATA, anti-Th/To, anti-RNA polymerase III, Ro52/TRIM21), little is known about the prognostic value of the other antibodies associated with SSc or UCTD.

## AUTOANTIBODIES IMPLICATED IN THE PATHOGENESIS OF EARLY SYSTEMIC SCLEROSIS

Achieving a fuller understanding of the pathogenesis of early SSc may help identify early effective therapeutic options to stop or slow progression of SSc and related organ complications. One of the barriers to understanding how autoantibodies to intracellular targets could be pathogenic was the conceptual challenge of how SSc autoantibodies, especially ANA, could penetrate living cells and bind to their cognate target and then participate in the pathogenesis of the disease. Many of these conceptual concerns have been allayed with the understanding that various forms of cell death [i.e. apoptosis, necrosis, NETosis (NET, Neutophil Extracellular Trap), pyroptosis] [49,50] as well as moonlighting macromolecules [51] may provide the opportunity for autoantibodies to bind to their intracellular targets and impact on inflammatory and other pathogenic pathways [4].

An increasing spectrum of autoantibodies is being proposed as having potential pathogenic roles in the initiation and progression of SSc vasculopathy and fibrosis [4]. Referred to as ‘functional autoantibodies,’ they have been detected in 26–100% of SSc patients and include antiendothelial cell antibodies, antiplatelet-derived growth factor receptor, anti-AT1 receptor, endothelin-1 type A receptor, and interferon-inducible protein 16 (Table 3) [3^▪^,^4^,^7^^▪▪^,^46^,^52^–^68^]. Functional autoantibodies are typically directed against nonnuclear and cell surface (i.e. receptors) targets, which are more easily accessible to circulating autoantibodies and may initiate tissue damage resulting in certain disease features [6^▪^,^69^]. One of the main limitations of studies of functional autoantibodies has been limited evidence of their genesis and presence in early SSc. Studies in the future should focus on early SSc rather than established disease if the significance of these autoantibodies and their potential link to therapeutic interventions is to be understood.

**Table 3:** Frequency and pathogenic role of functional autoantibodies in established systemic sclerosis

Functional antibody	% Frequency	Pathogenic role in SSc	Other clinical associations	Reviews and references
Antiendothelial cell antibodies	44–84	Activation of endothelial cell apoptosis and stimulation of proinflammatory and profibrotic cytokines release in the microvasculature. Mediate endothelial damage and dermal fibroblast activation	More severe disease manifestations, for example, vascular, perivascular, and pulmonary diseases such as PAH	[52,53,54]
Antifibroblast antibodies	26–58	Target glycolytic enzyme α-enolase and induce proadhesive and proinflammatory phenotypic changes in fibroblasts by upregulating ICAM-1 expression, IL-6 production, and enhanced U937 cell adhesion	Associated with antitopoisomerase I, prevalence of ILD and PAH	[55,56,57]
Antifibrillin-1	>50	Activate fibroblasts *in vitro*. Simulate release of TGF-β in extracellular matrix. Conflicting data regarding primary or secondary role in pathogenesis	Higher levels detected in certain ethnic groups	[58,59]
Anti-MMP-1 and 3	49–52	Inhibit MMP collagenase activity, thereby prevention degradation of excessive collagen and extracellular matrix components	Specific for SSc; correlates with degree of fibrosis in skin, lung, and renal blood vessels	[60, 61]
Anti-PDGF receptor	33–100	Activation of the PDGFR. Stimulation of reactive oxygen species and collagen production, and converting resting fibroblasts into activated myofibroblasts. Shown to induce skin fibrosis *in vivo*	Potential therapeutic target for therapies such as rituximab, nintedanib, imatinib, and nilotinib	[62,63,64,65]
AT1 receptor and endothelin-1 type A receptor	82–83	Induce TGF-β, vascular cell adhesion molecule-1, IL-8, and chemokine ligand 18. They work by increasing intracellular calcium and neutrophil transendothelial migration and reduce regenerative capacity of endothelial cells	Associated with early and severe disease, PAH, digital ulcers, and renal crisis, diffuse SSc, lung fibrosis. Predicts SSc-related mortality, PAH, response to therapy, and incidental DU	[7^▪▪^, 66]
IFI16	18	Enrichment of IFI16 in CD31-positive vascular endothelial cells from SSc biopsies and circulating progenitor cells	Majority (77%) had lcSSc, longer disease duration and decreased DLCO. Associated with vasculopathy/DU	[67,68]

AT, angiotensin; DLCO, diffusing capacity for carbon monoxide; DU, digital ulcers; ICAM-1, intracellular adhesion molecule-1; IFI16, interferon-inducible protein 16; IL, interleukin; ILD, interstitial lung disease; lcSSc, limited cutaneous systemic sclerosis; MMP, matrix metalloproteinase; PAH, pulmonary arterial hypertension; PDGF, platelet-derived growth factor; SSc, systemic sclerosis; TGF-β, transforming growth factor-β.

One of the major challenges of widespread validation and adoption of functional autoantibodies in a routine clinical setting are the protocols and technologies used to identify them. To date, simply detecting binding of the autoantibody to the specific target has not been reproducible. As only one example, although early evidence indicated that antibodies to platelet-derived growth factor receptor were a very common feature of SSc when measured in a functional assay, but in static immunoassays that detect only autoantibody binding, the results are quite different and less compelling [4]. Perhaps high-throughput technologies capable of measuring functional autoantibodies will help bridge that gap and when they do, a new era of diagnostics in SSc will unfold.

## CLINICAL CARE PATHWAY USING AN EARLY DIAGNOSIS OF SYSTEMIC SCLEROSIS AND AUTOANTIBODY TESTING

**FIGURE 1:**
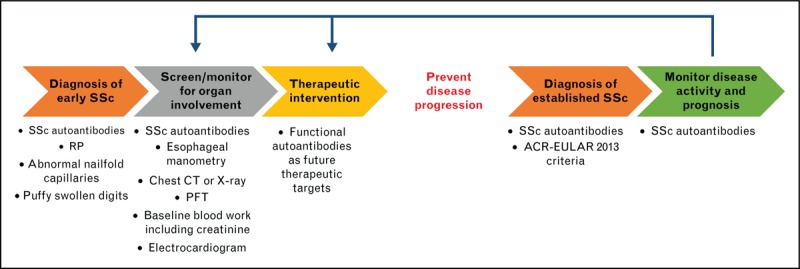
Clinical care pathway using an early diagnosis of SSc and autoantibody testing. ACR-EULAR, American College of Rheumatology-European League Against Rheumatism; CT, computed tomography; PFT, pulmonary function test; RNP, ribonucleoprotein; RP, Raynaud's phenomenon; SSc, systemic sclerosis. Adapted from [23].

There are potential applications for autoantibody detection in the care of SSc patients based on evidence that autoantibodies in SSc are predictors of disease development, useful for diagnosis and definition of disease endotypes, prognosis, and indicators of potential therapeutic targets. A clinical care pathway is a structured and standardized strategy of care (usually multidisciplinary in nature) for a defined population incorporating evidence-based guidelines into practice using an algorithm or protocol to guide care [70]. A proposed outline of a clinical care plan for evaluation and management of SSc with a particular focus on early diagnosis of SSc and the implementation of autoantibody testing is illustrated in Fig. 1.

When considering a clinical care pathway for SSc, it is important to begin intervening at the earliest phases of disease. As mentioned previously, there are several definitions of early SSc and both the ‘early SSc’ and VEDOSS criteria include the detection of SSc autoantibodies. Even for UCTD and patients with capillarscopic changes, the presence of autoantibodies is an important indicator for faster progression to established SSc. Hence, the presence of SSc autoantibodies early in the disease course may identify patients who require closer follow-up, thus preventing a delay in diagnosis and hence better outcomes. Early SSc patients should be assessed for organ involvement. Detecting antibodies associated with SSc-related organ manifestations early in the disease course can guide directed investigations and monitoring for end-organ involvement in a cost-effective manner. The early phase of SSc is also a window of therapeutic opportunity for altering disease progression and also initiating treatment prior to irreversible damage [20^▪^]. In the future, there may be interventions directed against functional autoantibodies and treatment may therefore be personalized to their autoantibody profile.

## CONCLUSION

There is compelling evidence that autoimmunity has important pathogenic, predictive, diagnostic, and prognostic relevance in SSc. In particular, autoantibodies are one of the earliest observable features of the disease, although more studies are needed to elucidate the presence and role of functional autoantibodies in early SSc. With the advent of more sensitive, multiplexed microarrays, more autoantibodies relevant to diagnosis and pathogenesis of SSc continue to be discovered. We have outlined a clinical care pathway that uses autoantibodies to help make an earlier and accurate diagnosis, monitor for disease development and progression, and potential therapeutic targets.

## Acknowledgements

The authors acknowledge the strong leadership of Drs Murray Baron and Marie Hudson at McGill University, Montréal, Québec, and the dedication of the Canadian Scleroderma Research Group. M.J.F. is a consultant to Inova Diagnostics Inc., (San Diego) and Werfen International (Barcelona, Spain).

### Financial support and sponsorship

None.

### Conflicts of interest

M.J.F. has received honoraria and/or gifts in kind (diagnostic reagents and kits) from Inova Diagnostics Inc. (San Diego, CA) and Euroimmun GmbH (Luebeck, Germany). M.Y.C. has no conflicts of interest.

## REFERENCES AND RECOMMENDED READING

Papers of particular interest, published within the annual period of review, have been highlighted as:

▪ of special interest
▪▪ of outstanding interest
